# Total Posterior Leg Open Wound Management With Free Anterolateral Thigh Flap: Case and Literature Review

**Published:** 2013-09-27

**Authors:** Soleiman Osman, Stephanie Chou, James Rosing, David E. Sahar

**Affiliations:** Department of Surgery, Division of Plastic and Reconstructive Surgery, University of California Davis Medical Center, Sacramento, Calif

**Keywords:** Achilles tendon, anterolateral thigh flap, free tissue transfer, microsurgery, surgical flap

## Abstract

Soft tissue coverage of the exposed Achilles tendon is a unique reconstructive challenge. In this report, we describe the management of a large posterior leg wound with exposed Achilles tendon using a free anterolateral thigh (ALT) flap. A careful review of alternative reconstructive options is included, along with their respective advantages and disadvantages. A 32-year-old white man suffered a fulminant right lower extremity soft tissue infection requiring extensive debridement of the entire posterior surface of the right leg. The resulting large soft tissue defect included exposure of the Achilles tendon. Reconstruction of the defect was achieved with an ALT flap and split-thickness skin graft for coverage of the Achilles tendon and gastrocnemius muscle, respectively. The patient was able to ambulate independently within 2 months of the procedure.

Large posterior leg wounds that involve an exposed Achilles tendon require a unique approach to resurfacing. The reconstruction trend shifted to free-tissue transfer in the 1980s because of unreliability of local tissue rearrangements and pedicled flaps in the lower third of the leg.^1^ Treatment demands sufficient mobility, durability, strength, protection from the friction of normal wear and tear, and resistance to infection.^2^ The use of microsurgical free-tissue transfer yields the highest reliability in soft tissue coverage and best long-term outcomes to date.^3^^,^^4^ It is generally regarded the highest rung of the reconstructive ladder among alternative procedures such as direct skin closure, split-thickness skin graft (STSG), and local-regional flaps.^3^ In this case report, we present and discuss the management of posterior leg open wounds with Achilles tendon exposure using an anterolateral thigh (ALT) flap and STSG. We also review alternative donor sites for free-tissue transfer for total posterior leg open wounds.

A number of flaps are useful in lower extremity coverage, including the reverse sural fasciocutaneous,^5^ latissimus dorsi myocutaneous (LDM),^6^ lateral arm fasciocutaneous,^7^ scapular/parascapular fasciocutaneous,^8^^,^^9^ free transverse rectus abdominis myocutaneous (TRAM),^10^^,^^11^ and the deep inferior epigastric perforator (DIEP) fasciocutaneous flap.^12^ While these flaps are described for soft tissue coverage of the Achilles tendon, they all have limitations in the successful reconstruction of these wounds. The ALT free flap for large posterior leg wound coverage of the Achilles tendon provides excellent tissue bulk, which includes skin/fascia, and has minimal donor-site morbidity. This flap does not sacrifice muscle (ie, LDM) and offers minimal potential motor nerve injury (ie, lateral arm flap). However, anatomical variability of intramuscular perforators makes this flap surgically complex and perhaps intimidating to surgeons who do not routinely raise this flap.

## CASE REPORT

**Figure 1:**
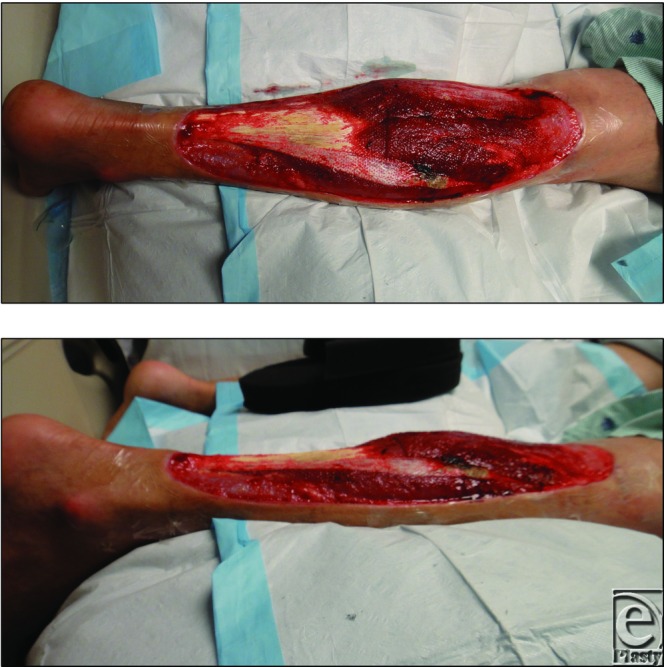
Right posterior leg wound approximately 15 × 40 cm^2^ encompassing the entire posterior surface of the right leg and Achilles tendon.

A 32-year-old white man presented to a local emergency department with a noticeable puncture wound, progressive swelling, and erythema to the right leg. The injury was sustained when he fell against the metal pedal of his bicycle. After 3 days of hospitalization and multiple courses of antibiotic, a surgical team was consulted for operative debridement of the wound. Diagnosis and confirmation of necrotizing fasciitis by polymicrobial group A streptococcal organisms was made. The resultant surgical debridement of the right posterior leg over the span of 1 week resulted in an open wound extending from the right popliteal fossa to about 8 cm from his right ankle (Fig 1). After a period of negative pressure wound therapy, the patient was referred to University of California Davis Medical Center for definitive reconstruction of his posterior right leg wound with exposed Achilles tendon. The patient had a history of Hodgkin's lymphoma, currently in remission, and ureteral stone causing hydronephrosis. He had no history of diabetes, alcoholism, or other general medical condition. The patient was a habitual smoker with history of drug abuse. Upon transfer to University of California Davis medical center, he was immediately prepared for free-tissue transfer.

### Markings and surgical anatomy

**Figure 2:**
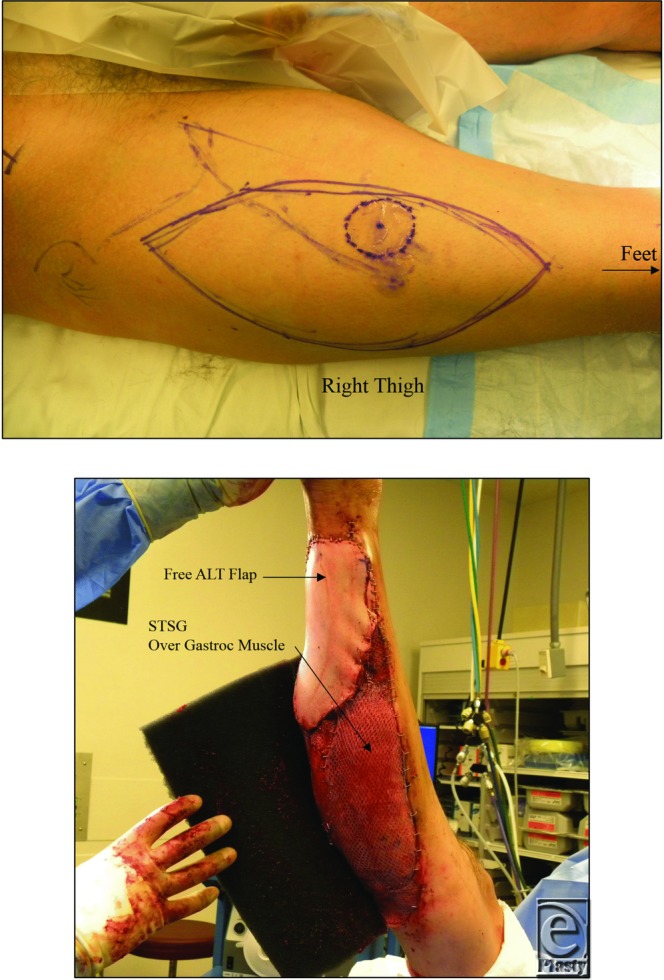
ALT Flap design, 15 × 20 cm^2^. The ALT flap was centered around an area mid-point between the anterior superior iliac spine and lateral patella. Cutaneous perforators were detected by a handheld pencil Doppler. The Achilles tendon was entirely covered with the ALT flap, while the remaining open wound was covered with skin graft.

A line was drawn from anterior superior iliac spine to the lateral border of the patella (Fig 2). This line roughly corresponds to the intermuscular septum between the rectus femoris and the vastus lateralis muscles. Skin vessels supplying the ALT flap are centered along this line or slightly lateral of it.^13^ The midpoint of this line, an area where skin perforators are generally located, was marked.^14^ Additional perforators were located within 10 cm proximal and distal to the midpoint perforator.^13^ At each location, this conventional naming system of skin perforator clusters provides a guideline for vascular localization.^13^ A handheld Doppler was used to localize the skin perforators in the anterolateral aspect of the thigh. The required size of the ALT flap was then measured and marked incorporating these perforators.

### Operative management

**Figure 3:**
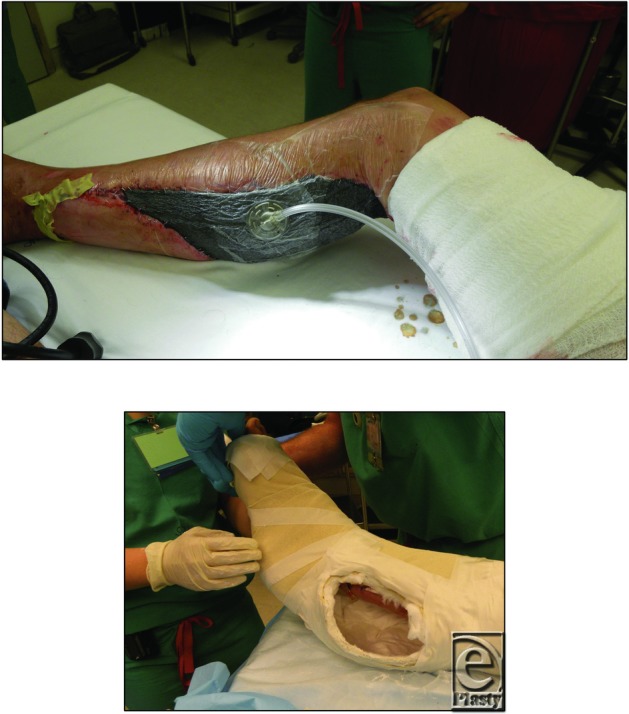
Postoperative care: The skin graft was placed under negative pressure and the flap was monitored with internal Doppler. The right leg was placed in a cast for 5 days with a window to monitor the free flap.

Examination of the foot revealed palpable dorsalis pedis and posterior tibial vessels and a normal modified lower-extremity Allen's test. After debridement of the open wound, the right posterior tibial vessels were exposed for use as recipient vessels. A large free ALT flap with a 15 × 20 cm^2^ skin paddle based on 2 perforators was harvested from the right thigh for coverage of the right Achilles tendon. No thinning of the flap was performed. Of note, the flap pedicle was medial and pierced through the medial aspect of the rectus muscle, slightly increasing dissection time. Once the flap was raised, the pedicle was divided at the bifurcation from the profunda femoral artery and was anastomosed to the posterior tibial vessels using a surgical microscope without complication. Vein couplers were used for venous anastomosis. Arterial anastomosis was performed with interrupted 9.0 Nylon suture in an end-to-end fashion. The flap was inset to completely cover the Achilles tendon. The remaining exposed gastrocnemius muscle was covered with STSG from the right thigh and vacuum-assisted closure therapy (KCI, San Antonio, Texas) was initiated (Fig 3). The foot perfusion was examined by normal capillary refill in all toes and palpable dorsalis pedis artery.

### Postoperative management

**Figure 4:**
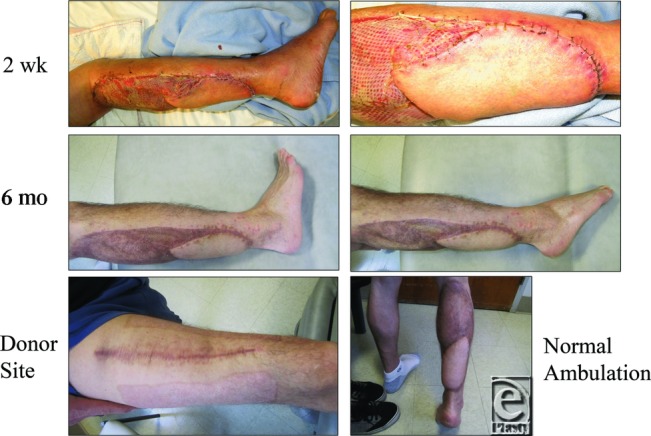
Patient was kept in the hospital for 6 days and discharge with outpatient follow-up. All wounds healed well and patient started independent ambulation by the end of second month.

**Figure 5:**
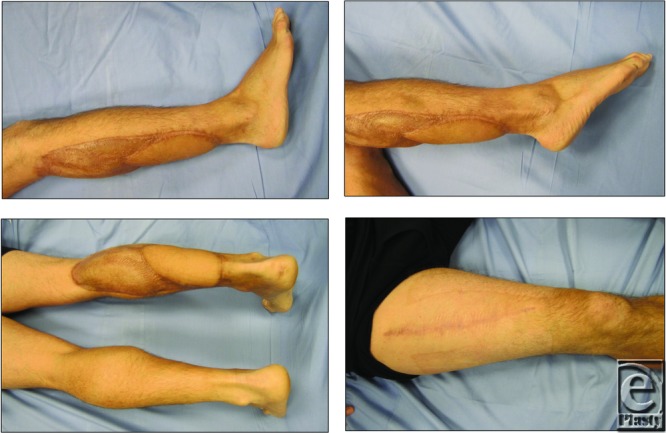
One year after the operation, patient had normal ambulation without any complaints.

The patient was placed in an intensive care unit for Cook Doppler monitoring for 4 days postoperatively and on a splint to keep his foot at 90 degrees. The Cook Dopplers and vacuum-assisted closure device were removed on postoperative day (POD) 5, and the patient was discharged home on POD 7 (Fig 4). Although the patient was scheduled for immediate physical therapy to improve foot function, he did not begin therapy until 4 to 5 weeks after discharge due to financial difficulties. Despite the late start, the patient was able to gain independent ambulation by 8 weeks postoperatively (Fig 5).

## DISCUSSION

Soft tissue coverage of the exposed Achilles tendon remains a reconstructive challenge. It demands the creation of a flap thin enough to allow normal footwear, but durable enough to permit tendon gliding, and to withstand the shearing forces of ambulation.^2^ Since the components of the immune system and antibiotics are carried to the wound tissue, it is imperative to maintain an adequate vascular supply to the soft tissue to minimize infection.^15^ Exposed Achilles tendon without paratenon may worsen bacterial contamination and cause tendon desiccation, thereby leading to necrosis and dehiscence of the tendon and local tissue.^15^^,^^16^ The goals for microsurgeons for repair of soft tissue defects overlying the Achilles tendon with free tissue transfers are to improve functionality, maintain appearance of the recipient and donor site, prevent donor-site morbidity, and minimize flap failure risk. Several different types of free flaps have been described in the literature to address tissue coverage of an exposed Achilles tendon with its own benefits and deficiencies.

### Limb salvage and amputation

The clinical utility of the lower extremity injury severity scores, including Mangled Extremity Severity Score^17^ and modified versions of it,^18^ should be used cautiously to determine the fate of a lower extremity.^19^ Their predictive power and guidance have been questioned in determining amputation.^19^^-^23 Bosse et al^24^ in the Lower Extremity Assessment Project determined prospectively that the functional outcomes of salvaged limbs were equivalent in 2-year outcomes to those of amputation when controlled for injury severity score. In a subsequent retrospective publication, Bosse et al^25^ found that initial plantar sensation was not a prognostic value for long-term plantar sensory status, nor of functional outcomes, and should not be a parameter for limb-salvage decision making. For large posterior wounds if infection is severe, affecting the bone in advanced-age patients, the decision between salvage and amputation requires consideration of many factors: vascular status, socioeconomic status, cost, availability of microsurgical reconstructive service, and compliance with and ability to return to work. Many surgeons regard the combination of posterior tibial nerve damage and severe trauma to the vascular supply and bone as contraindication for salvage.^26^ Studies indicate that salvage is widely preferred to amputation.^27^ After surgical treatment of lower-extremity injuries, O’Toole et al^28^ identified factors that determined patient satisfaction. Their satisfaction correlated more to function, pain, and the presence of depression at 2 years than by attributes of the patient, injury, or treatment.^28^ The Lower Extremity Assessment Project study specifies that positive results are affected more by a patient's socioeconomic status and personal resources than by initial treatment types.^29^ When amputation is unavoidable, microsurgical procedures exist, for example, fillet flaps, to cover the resultant amputation wound for preservation and lengthening the remaining stump, facilitate prosthesis use, and avoid other donor-site sacrifice.^30^^-^32 However, isolated Achilles tendon exposure wounds without tibial nerve or vascular injury are good candidates for reconstruction rather than for amputation.

### Granulation tissue

Referring institutions often rely heavily on the formation of granulation tissue over lower extremity wounds prior to reconstruction consultation. Waiting for this response can be detrimental to the patient requiring flap coverage. In Achilles tendon injuries, granulation tissue cannot substitute for specialized fascia, subcutaneous, or dermis tissue, nor can it replace other highly specific tissues (bones, cartilage, and tendons). In addition, granulation tissue is relatively inelastic and its adherence and potential tendon tethering can cause further dysfunction of the lower extremity.^33^ The prolonged immobilization of the ankle necessary to achieve granulation tissue could further hamper range of motion and may require longer physical therapy postoperatively. Debridement of an infection requires an assessment that necrotizing muscle cannot be concealed under granulation tissue. For flap coverage granulation tissue is unfavorable and consequential to the function, and debridement is recommended of all granulation tissue prior to coverage.^33^

### Skin grafts and local and perforator flaps

Skin grafting overlying the Achilles tendon is generally avoided due to (1) poor durability of the graft in this region, (2) inability of granulation tissue to form prior to grafting, and (3) possibility of tethering the tendon, thereby limiting ambulation.^34^ These factors can detract from the overall success of a skin graft over tendons. The plastic, orthopedic, and podiatric literature does not generally advocate skin grafting over the Achilles tendon as a reconstructive option.^34^ Skin grafting has been proposed as a potential option over the Achilles tendon^34^; however, the outcome, even with adjunct therapies, proves not as effective as free-tissue transplantation. With skin grafting, there is a high risk of recurrent ulceration. Skin grafting is not a long-term solution for the ambulatory population; and so skin grafting of the Achilles tendon cannot be regarded as a routine treatment.

Local flaps are generally not used for Achilles reconstruction because of inadequate tissue coverage, although local perforator flaps could potentially be used for this purpose. Local perforator flaps may be adequate for only small wounds over the Achilles tendon due to relative paucity of soft tissue in the lower third of the leg. The relative success of free-tissue transfers obviate the need for such local options in large open wounds.^35^

### Sural artery fasciocutaneous flap

The sural artery flap is a fasciocutaneous skin island flap supplied by the vascular axis of the sensitive superficial nerves; the fasciocutaneous flap was first described by Ponten^36^ as a random plexus without identifying the artery penetrating the deep fascia that enters the plexus.^37^ Masquelet et al^38^ describe the concept of the neurocutaneous flap using accompanied arteries of the cutaneous nerves^37^ and the anatomy and clinical indications of its use for reconstruction of soft tissue defects of the distal third of the leg.^39^

The distal sural artery flap for coverage has been popular^39^ and reported with dimensions as large as 10 × 16 cm^2^ based on several advantages,^40^ making this flap a good alternative to microsurgical reconstruction in many cases to cover the defects of the lower third of the extremity. Advantages of this flap are as follows: (1) it is a thin fasciocutaneous flap with good soft skin contouring, (2) operative technique is simple and fast, (3) under regional anesthesia, (4) direct closure of the donor area is possible for small flaps, and (5) major arteries or nerves are not sacrificed.

While it is an alternative to microsurgical reconstruction, sural flap's shortcoming is lack of robust blood supply due to source of vascularity from peroneal perforators at the ankle. Partial or complete necrosis has been reported in up to 36%,^39^ particularly in high-risk, critically ill, older patient populations and in flaps exceeding 9 × 12 cm^2^ in dimension.^41^ Large posterior wounds and wounds crossing the vascular axis of the sural fasciocutaneous flap laterally are contraindications to this procedure. This flap generally cannot be closed primarily with harvests exceeding 4 cm in width.^41^

### Free transverse rectus abdominis myocutaneous flap

The rectus abdominis is a type III muscle in Mathes and Nahai classification receiving blood supply from 2 dominant pedicles: superior and inferior deep epigastric vessels^42^; it can be raised as a muscle or musculocutaneous flap, the TRAM flap.^10^^,^^43^^,^^44^ Although, this flap was used successfully for lower extremity reconstruction in the past,^43^^,^^44^ its use in lower extremities is not considered a first choice due to risk of donor-site morbidity, abdominal hernia, and bulkiness of the flap. Furthermore, fasciocutaneous flaps have been shown to be as effective as musculocutaneous flaps in contributing to the sterilization and healing of an infected wound.^45^

### Latissimus dorsi myocutaneous flap

The Latissimus Dorsi Myocutaneous (LDM) flap is one of the most preferred donor sites for soft-tissue reconstruction of defects overlying the Achilles tendon; a flat, fanlike muscle flap that can be harvested up to 20 × 40 cm^2^,^46^ thereby providing sufficient coverage for areas of large injuries. The latissimus dorsi muscle is a type V muscle in the Mathes and Nahai classification.^42^ This flap may be based on either the dominant subscapular/thoracodorsal system or the more distally located secondary segmental paraspinous perforators.^47^ The LDM flap has a long 18-cm high-caliber pedicle, making microvascular anastomosis relatively easy, and can occur outside the zone of injury. A large skin paddle up to 14 × 25 cm^2^ over the muscle can be harvested along with this flap.^48^ The LDM flap has the advantage of providing ample soft tissue that exhibits consistent vasculature easily raised in a one-stage procedure.

Potential functional compromise at the donor site and difficulties positioning the patient in a lateral decubitus pose during tissue harvest can restrict the preference of an LDM flap. Russell et al^49^ described quantitative measures of shoulder weakness in 19 of 23 patients who had undergone harvesting of the latissimus dorsi flap; however, the functional limitations are relatively minor and are minimized by the recruitment of other muscles.^49^ The dysfunctions are infrequent and should not deter from raising this flap. Other disadvantages of the LDM flap include bulkiness and poor aesthetic outcome due to potential skin grafting of the donor site when primary closure is not possible. Bulkiness may be less of an issue due to atrophy of the muscle over time. Contraindications of the LDM flap include a patient's inability to be positioned on their side, severe comorbities, as well as a patient's history of previous operations disrupting blood supply from a posterior thoracotomy.

### Lateral arm fasciocutaneous flap

The lateral arm flap consisting of skin, fat, and fascia is supplied by the septocutaneous branches of the posterior radial collateral artery, develops from the profunda brachii, and is reported to have a reliable and consistent vascular anatomy.^50^ The lateral arm flap bears good tendon gliding due to adequate soft tissue, yet it is not bulky enough to affect cosmesis and function. Although the skin territory can be as large as 12 × 18 cm^2^ based on injection studies and reports,^51^^-^54 flaps should be located within the “zone of security” extending 12-cm proximal to the lateral epicondyle and including one third of the circumference of the upper arm.^55^

An unfavorable distinction of the lateral arm flap is possible functional impairment of the donor site due to the intricacy in dissecting the pedicle. The deep positioning of the proximal segment of the profunda brachii artery underneath the lateral head of the triceps muscles and its close relationship to the radial nerve renders dissection of this vascular pedicle more challenging. When a longer pedicle is required, extensive dissection onto the brachial vessels may lead to weakness in the arm being operated on, risking injury to the radial nerve. Further transection of the triceps head may lead to reduced strength and limited extension of the lateral arm, while loss of sensation at the proximal and posterior regions of the forearm has been observed. Assessing complications and morbidity of the donor sites, Graham et al^54^ reported that among the 123 lateral arm flaps he has operated on, 27% of patients reported dissatisfaction with the appearance of the donor site. Furthermore, 19% reported elbow pain, 59% reported numbness in the forearm, 17% reported hypersensitivity to stimuli such as cold or vibration, and 83% reported the flap to be bulky. The lateral arm flap can also be used as an osteocutaneous flap as well as a neurosensory flap using the lateral brachial or cutaneous nerve, a branch of the radial nerve. The neurosensory feature is more important in covering areas of the foot, which are weight bearing, similar to areas of the hand, and much less important in covering the Achilles tendon.

### Parascapular/Scapular fasciocutaneous flap

Another upper extremity alternative to the latissimus dorsi flap in Achilles region reconstruction is the parascapular flap; a fasciocutaneous flap perfused by the descending cutaneous branch of circumflex scapular artery. The flap has been harvested up to 15 × 25 cm^2^ on a single pedicle^56^^,^^57^ and is relatively simple to dissect due to its consistent vasculature and high caliber. The parascapular flap is exceptionally durable due to its thick dermal layer, which makes it more resistant to compressive loading, thus providing for better foot coverage. Other advantages of this flap include hairlessness of the skin and a similarity in texture and color to that of facial skin providing a more satisfactory appearance. Large-size parascapular flaps allow for easier wound closure and leave less conspicuous scarring.^58^

Similar to the lateral arm flap, harvesting of the parascapular flap may result in potential functional compromise of the upper extremity. Physiotherapy for 2 to 3 weeks is typically required in patients who underwent harvesting procedures of this flap.^55^ The flap is often deemed too bulky and may result in significant scarring at the donor site if tensionless wound closure is not possible.^55^ When a long pedicle is needed, dissection is tedious and difficult as the surgeon is required to work through the posterior triangle where the circumflex scapular artery passes between teres major, teres minor, and the long head of the triceps muscle. Transection of muscles in this triangular region can significantly impair functions, leading to shoulder weakness and a limited range of motion.^59^ In addition, when bringing the patient in a prone or lateral decubitus position, extra care must be taken to prevent injury to the brachial plexus.

The scapular flap, described by Dos Santos^60^ is a versatile cutaneous flap, but it is unsuitable for covering defects greater than 10 × 16 cm^2^ because of the limited vascular territory of the transverse branch of the circumflex scapular artery.^9^ A detailed study of the anatomy and dissection are available by Mayou et al.^61^ Its suitability has been proposed for foot defects^62^ and recently the scapular flap has been effectively used in Achilles tendon coverage, but most patients required thinning for normal footwear.^35^

Koshima and Soeda^9^ described the combined scapular and parascapular cutaneous flap for a wide defect of the lower leg. The elevated flap measured 13 × 30 cm^2^ and transferred for a postburn scar.^9^ The flap can be used successfully but the main disadvantage is that the secondary defect requires closure under significant tension with STSG, with the potential of making the scar unacceptable, especially in women, and unsuitable for coverage requiring sensory innervations.^9^ However, the combined flap based on the subscapular artery system is gaining popularity.^8^^,^^9^^,^^63^

### Deep inferior epigastric perforator fasciocutaneous flap

The DIEP flap is an abdominal construct; a skin and adipose flap, supplied by the deep inferior epigastric artery branching from the external iliac artery. Advantages of the DIEP flap include the ability to provide a large amount of skin and soft tissue, the potential to undergo thinning, low risk of weakness or herniation compared to the free TRAM flap, and more importantly it leaves the rectus abdominus muscle intact. A recent retrospective review of 475 free flap reconstruction patients, by Wan et al,^64^ reveals abdominal bulging and hernia to be 11.3% in free TRAM flaps and 3.5% in DIEP flaps. Harvesting demands a surgeon with significant microsurgical experience and requires a prolonged operative time, which precludes many patients with multiple medical conditions. The dissection of the vascular perforators of the DIEP flap is a challenge because it can be difficult to identify and often run close to the inscriptions of the rectus muscle. The flap may be too bulky for lower extremity coverage and may require further thinning for use in the obese-prone Western population. The surgical outcome of the DIEP flap is variable, as fat necrosis may occur due to the flap's lack of a robust blood supply. Granzow et al^65^ reported a 13% occurrence of fat necrosis among patients who underwent DIEP flap harvesting for breast reconstruction. Gill et al^66^ noted in a 10 year study approximately 5% of seroma formation, 12.9% fat necrosis and 0.7% of abdominal hernia in patients who underwent the same procedure.

### Anterolateral thigh fasciocutaneous flap

An underutilized construct for soft tissue reconstruction of the Achilles region is the ALT flap, a highly versatile flap that can be harvested either as a cutaneous, fasciocutaneous, or myocutaneous flap. The versatility of the ALT flap with a vascularized fascia lata combines Achilles tendon functional repair and soft tissue coverage in one stage. This composite ALT flap with vascularized fascia lata is rolled to serve as the tendon graft.^67^ The ALT composite flap is functionally reliable, esthetically pleasing, and successful in elderly patients as well.^68^

The ALT flap can be harvested as a sensate flap using the lateral femoral cutaneous nerve, and 2 other nerves that have a role, the superior perforator nerve and the medial perforator nerve.^69^ Different innervations allow diverse sensate combinations of ALT flaps; a smaller flap by sparing the lateral femoral cutaneous nerve, utilizing the superior perforator nerve and/or the medial perforator nerve, a bilobed harvest for dual innervation is possible or alternatively all 3 nerves for a larger flap. The sensibility of the ALT flap is important in heel reconstruction because it provides excellent tissue bulk allowing normal ambulation with normal footwear, in contrast to previous management, which required orthotics and prosthetics to maintain proper foot alignment relative to the ground in soft tissue problems with calcaneal fractures.^70^ Sensitivity also minimizes damage to the weight bearing area of the heel during the gait cycle by facilitating softer heel strike.

The flap is supplied by the descending branch of the lateral femoral circumflex artery and can be primarily closed when the flap does not exceed 9.5 cm width range.^71^ However, absolute measurements generally do not factor individual patient variability; therefore, ALT donor sites are primarily closed when flap width-to-thigh circumference ratio is less than 16%.^72^

Flaps 25 × 35 cm^2^ on a single dominant perforator have been used,^73^ but this may require skin grafting of the donor site thus leading to less esthetically pleasing results.^72^ The ALT flap has the advantage of providing ample subcutaneous tissue that allows for tendon gliding and high flexibility and can be harvested in large or irregular shapes. The tissue receives a reliable blood supply from perforators, and revascularization of recipient vessels is possible through harvesting the flap in a flow-through manner. Other advantages of the ALT flap include potential thinning, good skin quality, and the possibility to raise the flap under epidural anesthesia.^74^ It has been effectively demonstrated in a prospective study that because of the excellent blood perfusion, ALT perforator flaps have a beneficial outcome in the treatment of complex infected wounds of the lower extremity^45^; As an added bonus, ALT flap avoids the morbidity of a muscle flap. Free-tissue transfers across different publications illustrate that ALT outcomes are comparable to other studied free-tissue transfers in the treatment of lower extremity wounds.^7^^,^^8^^,^^12^^,^^35^^,^^54^^,^^66^^,^^75^^-^79

The ALT flap has been extensively used in Asia for complex Achilles reconstruction but has not found such popularity in the United States. Microsurgeons in the United States are often reluctant to harvest the ALT flap due to its variable anatomy and the preconception of the flap being thicker in the comparatively obese Western population.^14^^,^^80^ Sensory loss in the distribution of the lateral femoral cutaneous nerve was reported in 84% of patients in the study of Hanasono et al.^71^

### ALT anatomical vascular variations

The anatomical vascular variations encountered with the ALT flap are classified into 2 types: (1) variation in the course of the vessels supplying the skin and (2) variation of the vascular pedicle of the flap.^13^^,^^77^^,^^78^^,^^81^^-^83 The first is detailed in the literature, and the last is much less appreciated.^77^^,^^83^^-^87 Once these anatomic uncertainties of the ALT flap are understood, the flap can reliably be harvested. A large percentage of patients have at least 2 perforators, which allow a surgeon to readily choose the best. The uncertainties in the anatomical variations of the ALT flap perforators can be problematic to many microsurgeons.

Although the ALT was initially described as a septocutaneous perforator flap, the majority (88%) of the perforators are musculocutaneous.^88^^,^^89^ Dissection of musculocutaneous perforators is potentially difficult and tedious because it involves dissecting out perforators traversing the muscle layer and significantly prolongs surgical time. Extra care must be taken as transection of the vastus lateralis muscle may impair motor function, specifically affecting knee and ankle stability. Kuo et al^68^ noted a 30% and 40%, respectively, deficit in the isokinetic concentric measurements of dorsiflexion and plantar flexion, and a 10% to 25% deficit of quadriceps femoris muscle contraction forces in 2 patients who underwent reconstruction of the Achilles region with a composite ALT flap. There were no difficulties in daily ambulation noted.

### Preoperative imaging of ALT flap

There is no substitute for good surgical planning and technique. Currently, the hand Doppler approach has been recognized as the standard method used for vascular mapping in the planning of the ALT flap. While the device can be used in both a preoperative and intraoperative setting, offering several advantages such as noninvasive, small size, low cost, and portability, it provides very limited information on the size/quality of perforators and can only detect perforators of a certain depth. Recent advances show that computed tomographic angiography (CTA) and magnetic resonance angiography (MRA) better approximate the course of vessels by showing 3D images.^90^ However, because of the high cost, the MRA is often not the preoperative test of choice. Recently, the development of SPY imaging has been shown to aid in localizing perforators by using laser light source and fluorescent dyes to produce real-time images of cutaneous vessels. SPY imaging can provide flap perfusion and vessel caliber determination.^91^ However, because of limited depth of detection, this technique may be of limited use in obese patients and those with tortuous and long perforators. Preoperative implementation of MRA, CTA, or SPY is not regarded as routine at this time because of cost, time, and the inherent limitations of each imaging system.

### Timing of coverage

There is evidence that early soft-tissue coverage within 72 hours (<72 hours) provides better outcome for the patient in contrast to reconstructions that were delayed (>72 hours).^92^ However, early reconstruction is not always possible in trauma settings due to logistical constraints, concurrent injuries requiring more urgent attention, and the need for debridement. Delayed lower extremity reconstruction using free flaps has been shown to be safe, with good predictable outcomes.^93^ Aggressive debridement, coordinated microsurgical planning, and anastomosis outside the zone of injury are important factors that contribute to a successful result with delayed lower extremity reconstructions.

### Donor site scar preferences studies

Although it has been suggested that the ALT flap donor site is not a preferred choice for women, it has been shown in the Yu study,^14^ that female patients accepted the scar very well both preoperatively and postoperatively. In another study, Yeung et al^94^ discovered that the ALT flap donor site was the most preferred donor site, followed by the proximal lateral calf, and lateral arm donor site. Brown et al^95^ found similar results with patients who preferred the upper thigh donor site in head and neck reconstruction, and Kimata et al^96^ reported that between the radial forearm donor site and ALT site, 90.6% preferred the latter. Patient donor site preference correlates with the ability to conceal the scar.^94^ When an option in free flap selection exists, it is important to factor patient's desire to conceal the scar. The ALT flap leaves a relatively concealed donor site scar under clothing, and so is a good option for patients who are concerned about donor site scarring.

## CONCLUSION

The ALT fasciocutaneous flap is an excellent choice for large soft tissue defects overlying the Achilles tendon; it offers durable coverage while producing minimal donor site morbidity. Whereas many good options for free-tissue transfers exist for coverage of exposed Achilles tendon, the ALT flap should be considered among them. The donor-site is well tolerated and the flap is well suited to cover a large exposed tendon without paratenon. Although mostly musculocutaneous perforators make dissection of the vessels tedious, a good understanding of the anatomy and adequate training minimize surgeon anxiety and flap loss. As the success rate of ALT is approaching 100%, functional and cosmetic outcomes are increasingly becoming important topics.
